# Reversible Watermarking for Electrocardiogram Protection

**DOI:** 10.3390/s25072185

**Published:** 2025-03-30

**Authors:** Pavel Andreev, Anna Denisova, Victor Fedoseev

**Affiliations:** Geoinformatics and Information Security Department, Samara National Research University, Samara 443086, Russia; pa.andreyev@gmail.com (P.A.); denisova_ay@ssau.ru (A.D.)

**Keywords:** prediction error expansion, reversible contrast mapping difference expansion, integer transform-based difference expansion, compression-based watermarking, watermark capacity, PSNR, inter-lead relationship

## Abstract

The electrocardiogram (ECG) is one of the widespread diagnostic methods used in telemedicine. However, in the telemedicine systems, the data transfer process to the end user may suffer from security risks. Reversible watermarking can preserve the security of electrocardiograms and keep their original precision for correct diagnostics. In this paper, we present an extensive investigation of four reversible watermarking methods: prediction error expansion (PEE), reversible contrast mapping difference expansion (RCM), integer transform-based difference expansion (ITB), and compression-based watermarking. We discover new facets of the existing ECG watermarking methods (PEE and compression-based watermarking) and adapt image watermarking methods (RCM and ITB) to ECG signal. We compare different kinds of prediction and compression methods used in the studied methods and provide a watermark capacity comparison for different methods’ implementations. The research results will help in watermarking method selection in practice.

## 1. Introduction

An electrocardiogram (ECG) is an important diagnostic method for detecting deviations from normal heart rhythm, identifying myocardial damage, and other heart problems. In recent years, doctors, medical staff, and patients often store and exchange ECG data via the internet and various information systems to simplify and speed up data transfers. However, this exchange introduces security risks that can lead to data leakage or malicious changes to ECGs. Therefore, medical information systems must implement data protection mechanisms for ECG data.

Our paper focuses on ECG data integrity protection using watermarking methods. Watermarking is a process of imperceptibly embedding information into a host signal. This embedded information is called a watermark. If the host signal is subject to unauthorized changes, the data recipient cannot extract the watermark and therefore knows that the data have been corrupted. The destruction of a watermark after host signal changes is typical of fragile and semi-fragile watermarking methods, which are the focus of our research concerning ECG data integrity protection.

Based on the watermark extraction procedure, watermarking methods are classified into blind, semi-blind, and informed methods. Blind extraction does not use any information about the embedded watermark or the original host signal for extraction. Semi-blind methods may use additional information, for example, a secret key, but do not use the host signal for watermark recovery. Informed methods require knowledge of the host signal in addition to side information and the watermarked signal. In practice, blind and semi-blind extraction are preferable because the recipient may not know the host signal. Therefore, we focus on blind and semi-blind watermarking methods.

While watermarking introduces minor changes to the signal, precise knowledge of the host signal is critical for some applications. Watermarking methods that allow host signal restoration are called reversible. Since reversible watermarking is preferable for medical data protection, we chose it as the focus of our research.

Several works on ECG watermarking have been proposed in recent years. Most introduce non-reversible, blind, semi-fragile watermarking schemes based on wavelet transform [1,2,3,4,5,6,7,8,9,10].

Kumar et al., in [1], presented ROSEmark. This algorithm decomposes each ECG record into segments. Each segment is transformed using a three-level stationary wavelet transform (SWT), and the resulting approximation vector is subjected to a discrete cosine transform (DCT). Next, an extreme learning machine modulates the low-frequency coefficients, and the watermark is embedded in these modulated coefficients.

Khaldi et al. [2] proposed a frequency-domain watermarking method in which the signal is transformed into a 2D image and then subjected to an integer wavelet transform (IWT). Finally, the acquired coefficients are treated through Schur decomposition, and the watermark bits are integrated by altering the least significant bit (LSB) of the generated eigenvalues. A similar approach involving converting a 1D-ECG signal to a 2D-ECG image is applied in [3]. The resulting image is then transformed using wavelet decomposition for subsequent watermarking.

In contrast, refs. [4,5] use one-dimensional discrete wavelet transform followed by irreversible watermark embedding. In [6], an ECG watermarking technique based on redundant discrete wavelet transform (RDWT) and singular value decomposition (SVD) is developed. Duy et al. [7,8,9] embed watermark data by modifying the mean modulation relationship of approximation coefficients in the wavelet domain. While the experiments are conducted using EEG data, the method supports various biomedical time series data.

Only a few articles [11,12,13,14] describe reversible watermarking methods. Bhalerao et al., in [11], proposed an ECG watermarking scheme based on the prediction error expansion (PEE) approach. In PEE, watermarked data samples have doubled prediction error, where prediction error is the difference between the actual sample value and its approximation using neighboring samples. Because the neighboring samples remain unchanged during the watermarking process, the original value of the watermarked data sample can be retrieved precisely. Bhalerao et al., in [14], presented a method combining a deep neural network (DNN) and PEE for reversible watermarking. They used a DNN to improve prediction error computation. Unfortunately, reversible watermarking approaches based on expansion algorithms other than PEE have not yet been adapted to ECG signals. In [12], Tripathi et al. suggested a compression-based watermarking method for ECG data. This method applies even–odd embedding and the Fourier decomposition method to remove redundant components present in the host signal. Despite the compression used, this method allows approximate host signal restoration with improved signal-to-noise ratio characteristics. Kumar et al. [13] proposed a similar method based on discrete wavelet transform compression. However, existing compression-based ECG watermarking methods concern only lossy compression and provide only approximate recovery of the host signal.

The aforementioned research demonstrates that existing reversible watermarking methods for ECG signals cover a limited range of possible approaches. Our research aims to provide a comprehensive comparison of four reversible watermarking methods related to different approaches originally developed for image watermarking: reversible contrast mapping difference expansion (RCM) [15], integer transform-based difference expansion (ITB) [16], PEE, and lossless compression-based embedding (LCB). As only PEE has an existing implementation for ECG signals, we implemented RCM, ITB, and LCB for ECG data. We investigated different types of prediction algorithms used in PEE and different lossless compression methods for the LCB approach. The combination of reversible watermarking approaches and prediction/compression algorithms constitutes a set of method modifications compared in our research. For each algorithm modification, we studied the peak signal-to-noise ratio (PSNR) and watermark capacity properties. Finally, we summarize existing performance trends among the considered algorithms.

## 2. Materials and Methods

### 2.1. Data Description

Electrocardiography is a method for studying and recording the electromagnetic fields generated by cardiac activity. In this procedure, healthcare professionals place electrodes on the patient’s body to measure temporal variations in electrical potential between electrode pairs. The result is an electrocardiogram (ECG), a set of signals recorded during the procedure. Each signal represents a time series of electrical potential values or differences between specific electrode pairs.

Several electrode configurations exist for ECG measurements, with the 12-lead system being one of the most widely used; it employs 10 electrodes [17]. The 12-lead ECG system consists of 6 limb leads (I, II, III, aVR, aVL, aVF) and 6 precordial leads (V_1_ to V_6_), offering comprehensive cardiac electrical activity monitoring across frontal and horizontal planes [18]. The bipolar limb leads (I: right-to-left arm; II: right arm-to-left leg; III: left arm-to-left leg) and augmented unipolar limb leads (aVR, aVL, aVF) capture frontal plane vectors, while the precordial leads (V_1_: 4th intercostal space, right sternal border; V_2_: 4th intercostal left sternal; V_3_: midpoint V_2_–V_4_; V_4_: 5th intercostal midclavicular; V_5_: anterior axillary line; V_6_: midaxillary) provide horizontal plane insights. This configuration can be used to diagnose arrhythmias, ischemia, and structural anomalies.

ECGs are stored in digital files, and each file consists of leads corresponding to recorded signals.

Among the most popular ECG file formats are EDF (European Data Format) and WFDB (WaveForm DataBase). An EDF file includes a file header, lead headers, and lead data. The file header contains metadata such as the creation date, medical staff name, and patient data. Each lead header provides information such as the lead label, duration, number of samples, measurement unit, electrode data, and filter data. Additionally, the lead header contains the information required to reconstruct original physical signal values from the digitized ones. The lead data is stored as a series of two-byte signed integers.

In the WFDB data format, each ECG lead corresponds to a set of files (.hea, .dat, .atr). The “.hea” files are lead headers, the “.dat” files contain binary lead data, and the “.atr” files contain lead metadata. WFDB may use different encodings for lead information, storing between 8 and 32 bits per data sample. In our research, we focus on embedding a watermark into the lead data in ECG files.

### 2.2. General Reversible Watermarking Scheme

**Figure 1:**
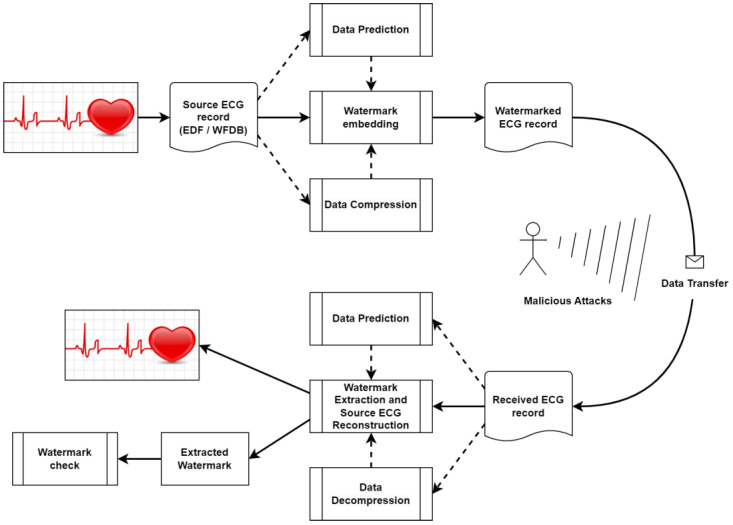
Reversible watermarking scheme.

An overview of the general reversible watermarking scheme is shown in Figure 1. We consider four reversible watermarking methods based on data prediction and data compression as key steps in the watermark embedding process. The PEE, RCM, and ITB methods employ some form of data prediction. Specifically, RCM and ITB use particular transformations to substitute samples with similar values, whereas PEE can utilize various prediction algorithms. The LCB method, on the other hand, uses data compression. Both lossless compression and prediction enable the reversibility of the watermarking process, ensuring that the original ECG signal can be fully reconstructed during the watermark extraction phase.

We assume that an intruder could tamper with watermarked ECG files during the data transfer process. If any changes are made to the ECG lead information, the watermark test will fail; otherwise, the original ECG signal will be precisely reconstructed.

In the following subsections, we provide detailed descriptions of the reversible watermarking methods used in this paper.

### 2.3. Reversible Contrast Mapping Difference Expansion

The RCM method [15] operates with pairs of data samples. It embeds s watermark bits into each pair. $x_{1},\;x_{2}$ are the samples of the host ECG signal. The following formula describes the relationship between the original samples $x_{1},\;x_{2}$ and their intermediate transformation $y_{1},\;y_{2}$:

(1) $$\left\{\begin{array}{c} y_{1}=\left(k+1\right)x_{1}-kx_{2},\\ y_{2}=\left(k+1\right)x_{2}-kx_{1}, \end{array}\right.$$

where $k=2^{s-1}$.

It is evident that the difference $y_{1}-y_{2}$ always has a zero remainder when divided by $\left(2k+1\right)$. To embed an integer watermark value $w\in \left[1;2k\right]$, the following transformation is used:

(2) $$y_{1}^{w}=\left\{\begin{array}{c} y_{1}+w,\;\;\;w\leq k;\\ y_{1}+w-2k,\;otherwise.\; \end{array}\right.$$

Finally, the pair of samples $y_{1}^{w},y_{2}$ substitutes $x_{1},x_{2}$ in the watermarked signal. It can be shown that $y_{1},y_{2}$ are close to $x_{1},x_{2}$ when the absolute value of the difference $x_{1}-x_{2}$ is small. As the ECG signal changes smoothly, subsequent samples have a relatively small difference. Thus, $x_{1},x_{2}$ are usually defined as subsequent samples.

If the numbers $y_{1}^{w},\;y_{2}$ are out of the suitable range of digital signal, the corresponding pair of samples has to be specially processed. To provide correct embedding in this situation, we offer to select $y_{1},\;y_{2}$ as such:

(3) $$\left(y_{1}-y_{2}\right)mod\left(2k+1\right)=0.$$

For example, we may assign $y_{2}{=x}_{2}$ and substitute $x_{1}$ for the nearest $y_{1}$ integer value which holds the aforementioned condition (3). Equation (3) signalizes that the pair of samples have to be omitted during extraction.

During the watermark extraction phase, for each pair of samples, the remainder of dividing $\left(y_{1}-y_{2}\right)$ by $\left(2k+1\right)$ is calculated. If the remainder is zero, there is no watermark embedded; otherwise, the value of remainder is a desired value of watermark bits. To reconstruct the original host signal samples $x_{1},\;x_{2}$, the following formula is used:

(4) $$\left\{\begin{array}{c} x_{1}=\frac{k+1}{2k+1}y_{1}+\frac{k}{2k+1}y_{2},\;\\ x_{2}=\frac{k}{2k+1}y_{1}+\frac{k+1}{2k+1}y_{2.} \end{array}\right.$$

Formulae (4) are suitable only for pairs of samples with successful embedding and if condition (3) is not satisfied. If condition (3) is met and $y_{2}{=x}_{2}$, the value of $x_{1}$ cannot be recovered in a single way. Thus, the RCM is not a completely reversible method. However, the absolute error of the $x_{1}$ reconstruction does not exceed 2k.

### 2.4. Integer Transform-Based Difference Expansion

The ITB method is based on a special integer transformation algorithm applied in [16] to frequency domain embedding for image data. This algorithm transfers sequence of integer numbers $x=(x_{1},\;x_{2},\;\cdots ,\;x_{n})$ into another integer sequence $y=(y_{1},\;y_{2},\;\cdots ,\;y_{n})$, where $y_{i}$ has the same parity for each i=1,…,n. The direct and inverse transformation formulae are the following:

(5) $$\begin{array}{l} y_{i}=2x_{i}-\left\lfloor \bar{x}\right\rfloor ,\\ x_{i}=\left\lfloor \frac{n\times y_{i}+\sum_{j=1}^{n}y_{j}}{2n}\right\rfloor , \end{array}$$

where $\bar{x}$ is a mean of the sequence x and $\left\lfloor \bar{.}\right\rfloor$ is a floor operation.

To embed watermark bits, we change the parity of the samples in transformed sequence y. The first sample remains unchanged to preserve information about the original parity of the sequence y. The embedding, extraction, and restoration are produced for samples with index i>1 by the following formulae:

(6) $$\begin{array}{l} y_{i}^{w}=y_{i}+w_{i-1},\\ w_{i-1}={(y}_{i}^{w}-p)mod2,\\ y_{i}=y_{i}^{w}-w_{i-1}, \end{array}$$

where $w_{i-1}$ is a watermark bit and $p=y_{1}mod2$ is a parity bit.

### 2.5. Prediction Error Expansion

Here, we regard the PEE algorithm version proposed in [11]. The key feature of the algorithm is a prediction of the sample used for embedding. The watermark bit is hidden into the difference between the predicted and original sample value.

Let us denote the original sample value as x, the predicted sample value as $\hat{x}$, and the watermark bit as w∈{0, 1}. Consequently, the signed sample value will be as follows:

(7) $$y^{w}=\hat{x}+2e+w,$$

where $e=x-\hat{x}$ is a prediction error.

The extraction and restoration formulae are as follows:

(8) $$\begin{array}{l} e^{w}=y^{w}-\hat{x},\\ w=e^{w}-2\left\lfloor \frac{e^{w}}{2}\right\rfloor ,\\ x=y^{w}-\left\lfloor \frac{e^{w}}{2}\right\rfloor -w, \end{array}$$

where $e_{w}$ is a prediction error for signed sample and $\left\lfloor .\right\rfloor$ is a floor operation.

It is evident that the distortion of the watermarked signal is as small as the prediction error. Thus, the main aspect of the PEE implementation is the prediction algorithm selection. In the present research, we consider several prediction methods described in Section 2.7.

### 2.6. Lossless Compression-Based Watermarking

LCB methods apply lossless compression techniques to reduce the storage space of the host signal. The freed storage space is then utilized to embed the watermark. The processes of watermark extraction and signal decompression allow for complete restoration of the original host signal. Existing compression-based methods often utilize discrete orthogonal transformations to compress only specific parts of the frequency information. In this study, we used only time-domain compression. To achieve this, we selected appropriate bit planes of the signal for compression and applied one of two lossless compression algorithms described in Section 2.7.4. The watermark was embedded in the remaining portion of the compressed bit planes.

### 2.7. Prediction Algorithms

PEE implementation requires prediction algorithms to be defined. We implemented three prediction methods: neighbor-based prediction, inter-lead prediction, and physical signal prediction.

#### 2.7.1. Neighbor-Based Prediction

In neighbor-based prediction, the neighboring samples of the host and watermarked signals are used for prediction. There are many ways to implement neighbor-based prediction. Here, we selected the prediction algorithm proposed in [11] because of its high efficiency. The algorithm calculates predicted value as follows:

(9) $$\hat{x_{i}}=\frac{y_{i-2}^{w}+y_{i-1}^{w}+x_{i+1}+x_{i+2}}{4}.$$

This type of prediction requires sequential embedding, starting with the third sample and ending with the third sample from the end of the signal. Additionally, the prediction uses signed sample values for the left-side samples and original values for the right-side samples. Thus, to preserve the same data values for prediction, the extraction is performed in right-to-left order.

#### 2.7.2. Inter-Lead Prediction

We propose predicting the lead for embedding by using the other ECG leads. Since all ECG leads describe the same process, such as the heartbeat, they must be correlated. Therefore, the samples from one lead can be used to predict the samples of another lead.

We hypothesize that each ECG lead can be predicted by a linear combination of the other leads. To define the coefficients of this linear combination, we suggest using linear regression [19]. This method corresponds to a modification of the PEE algorithm, wherein embedding is carried out only for one ECG lead while the other leads are used for prediction.

There are several options for the practical implementation of model training. We can train the model in advance using a large collection of ECG files and then use the prediction model for embedding and extraction. Alternatively, we can train the model on each specific signal. While this approach is more computationally intensive, it is also more accurate. However, an additional drawback of training on specific signals is that the model parameters need to be transferred along with the signal. Fortunately, the amount of additional information is small: 8 × (N + 1) bytes, where N is the number of leads used for prediction (in practice, N typically ranges from 5 to 11 leads). In this study, we implemented the second option—training the model for each ECG file—to demonstrate the possibility of achieving higher prediction accuracy.

#### 2.7.3. Physical Signal Prediction

Some of the six limb leads exhibit linear dependence on others due to the physical properties of the recorded signals [20]. Specifically, the relationships in (10) must be met.

In the physical signal prediction method, predictions are obtained as described in (10). In fact, these equations (or close to them) can be derived using linear regression, similar to the previous prediction method. However, the equations cannot entirely replace regression in all scenarios. For example, an ECG file may omit some linearly dependent leads. Moreover, the equations restrict the set of leads available for embedding to four (III, aVR, aVL, aVF), whereas regression allows embedding in any subset of leads.

(10) $$\begin{array}{l} III=II-I,\\ aVR=-\frac{I+II}{2},\\ aVL=\frac{2\times I-II}{2},\\ aVF=\frac{2\times II-I}{2}. \end{array}$$

#### 2.7.4. Compression Algorithms

ECG signals from different leads are usually highly correlated since they capture the same electrical activation process of the heart [21]. This inherent redundancy in multi-lead ECG data storage enables efficient deployment of compression algorithms for watermarking. We evaluated two lossless compression schemes optimized for binary data: run-length encoding (RLE) [22] and Huffman encoding [23]. Both algorithms reduce the original bit sequence length, freeing residual capacity in the bit plane structure for watermark payload insertion.

In addition to the mentioned algorithms, we also propose using lossless prediction error compression (LCBP) as an optional method. In this approach, the error between the predicted signal and the original host signal is compressed. Generally, compressing the error yields better results, allowing a larger bit sequence to be embedded into the signal.

It is crucial to select appropriate bit planes for compression and embedding. Lower bit planes do not compress well but result in minimal distortion of the signal’s form. In contrast, higher bit planes compress more effectively, but changes to these planes can lead to noticeable distortions in the watermarked signal. Therefore, the compression and embedding parameters must be carefully selected.

### 2.8. Data and Metrics

#### 2.8.1. Performance Metrics

To assess the quality of the watermarked signal, we exploit peak signal-to-noise ratio (PSNR). PSNR of distorted signal T with respect to original signal S is calculated as follows:

(11) $$\begin{array}{l} V_{MSE}=\frac{1}{n}\sum_{i=0}^{n-1}{\left|T\left(i\right)-S\left(i\right)\right|}^{2},\\ V_{PSNR}=10\log_{10}\left(\frac{S_{max}^{2}}{V_{MSE}}\right), \end{array}$$

where $S_{max}$ is a supremum of the original signal.

The watermark capacity is another criterion for evaluating the quality of a watermarking method. It is measured using the bits per sample (BPS) value, which is the ratio of the watermark bit length to the number of samples in the host signal.

#### 2.8.2. Database

In our experiments, we used a collection of ECG files described in [24]. This collection was obtained from the PhysioNet project database [25]. It includes 45,000 ECG files, each with a duration of 10 s. Each file contains records for 12 ECG leads with a sampling frequency of 500 Hz. For our research, we do not require such a large number of files; therefore, we selected 100 files from this collection.

**Figure 2:**
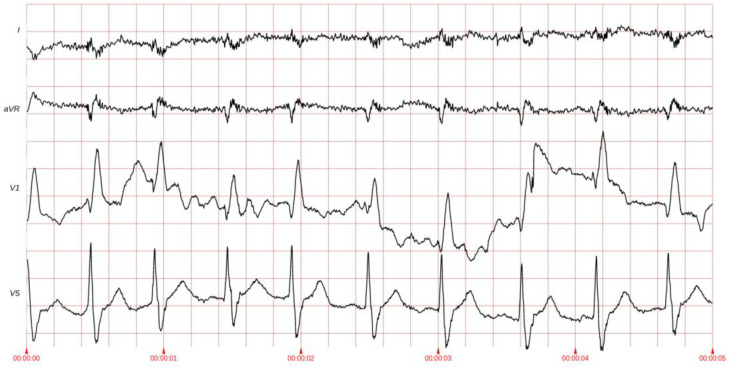
Example of ECG leads.

All ECG signals were acquired using an analog-to-digital converter with a sampling resolution of 0.001 mV (1 µV). The amplitude range (difference between maximum and minimum values within a single signal) exhibits significant variation both across patients and between different leads within individual patients. For the dataset analyzed, the mean digital amplitude range was 1548 digital units (equating to 1.548 mV), with a peak range of 5223 units (5.223 mV). We adopted the peak value of 5223 as the supremum for peak signal-to-noise ratio (PSNR) computations. Signals were stored in Waveform Database (WFDB) format files using a 16-bit storage depth, though the effective bit depth (utilized dynamic range) varied between 11 and 14 bits depending on lead positioning. Representative ECG waveforms from the dataset are illustrated in Figure 2.

## 3. Results

In this section, we present an experimental comparison of four reversible watermarking algorithms. We also discuss the selection of various prediction algorithms for PEE and the configurations for lossless compression algorithms. Finally, we evaluate the watermark capacity and quality of the target algorithms.

### 3.1. Prediction Algorithm Comparison

The PEE algorithm can be implemented in different ways, depending on the prediction algorithm used. We provide a comparison of seven prediction schemes for each ECG lead. Table 1 lists the prediction algorithms employed. The prediction algorithms from 1 to 3 are based on inter-lead relationships, either reconstructed through regression or described by physical relationships between signals. The prediction algorithms from 4 to 7 are variations of Formula (9), based on different numbers of neighbors.

To measure prediction quality, we applied the PSNR metric between the predicted and original signals for each ECG lead. A higher PSNR indicates better prediction.

**Figure 3:**
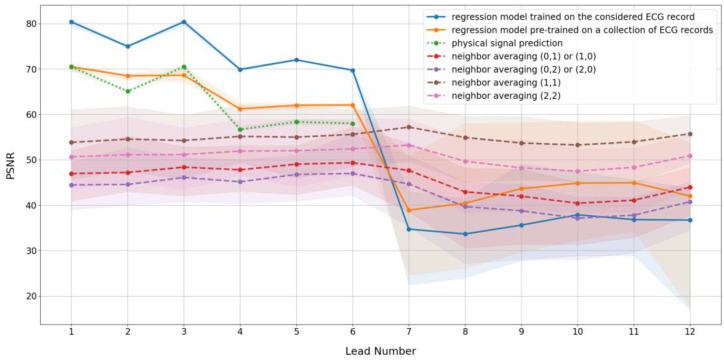
Prediction quality vs. lead number graph.

Figure 3 illustrates the PSNR values for various prediction algorithms and leads. Note that the colored backgrounds in Figure 3 represent the dispersion of values across the individual files used in the experiments, relative to the mean values shown by the lines.

For neighbor-based prediction, it is sufficient to use samples from both sides of the sample being predicted. Using predictions from the left and right sides yields noticeably lower PSNR. Regarding the number of samples used in the prediction formula, the two-neighbor, both-side prediction is better than the four-neighbor case. However, earlier research [11] indicated that four-neighbor-based predictions are optimal. The differing datasets used for testing may explain the variations in results when compared to [11]. Nonetheless, the two-neighbor and four-neighbor both-side predictions are similar and, therefore, may be used interchangeably.

Figure 3 demonstrates that inter-lead relationship-based prediction provides a larger PSNR for the first six (limb) ECG leads. This observation supports the idea that some of the limb leads are linearly dependent. Additionally, regression-based prediction performs better than prediction based on physical relationships. The reason for this is the implicit noise filtration provided by regression. Nevertheless, physical prediction has the simplest implementation and requires fewer computing resources than regression model training. A compromise between prediction quality and computational complexity can be achieved by using regression trained once on a collection of files. For the remaining six precordial leads, or in cases where the contents of the file do not correspond to the standard twelve leads, neighbor-based prediction is more effective. This method is universal and does not require a large amount of calculations.

### 3.2. Compression Algorithm Comparison

We consider RLE and Huffman compression algorithms for the LCB watermarking method. The RLE algorithm encodes the length of equal bit series using a B-bit number, where B is a parameter of the algorithm. The Huffman encoder has one parameter, L, which represents the length of the code symbol. Both parameters B and L must be selected experimentally. Another parameter common to the LCB watermarking method is the compressed bit plane index, P. The variations of B, L, and P determine the set of compression settings used in the experiment.

To measure compression quality, we computed the saved storage space using the following formula:

(12) $$V=1-\frac{C}{U},$$

where C is the storage space after compression and U is the storage space before compression. The bigger V corresponds to the higher compression.

The quality of the watermarked signal does not depend on the compression algorithm. It was measured using the PSNR value between signed and host signal.

**Figure 4:**
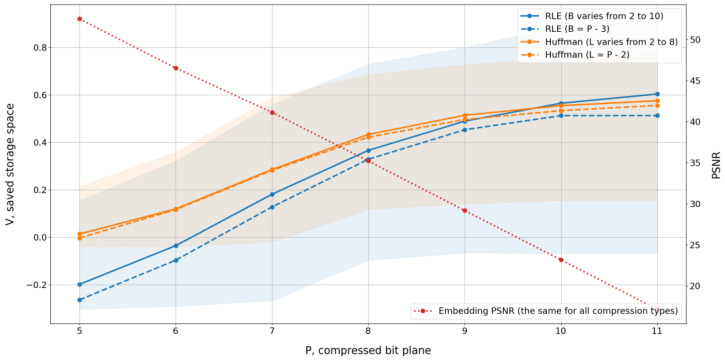
Saved storage space and PSNR values for watermarking in different signal bit planes.

Figure 4 shows the saved storage space and PSNR values for different bit planes used for compression and watermarking. Colored backgrounds have the same meaning as in Figure 3.

We used only bit planes from 5 to 11 for compression and watermarking because only these bit planes demonstrated a good compression effect and are therefore suitable for embedding. The numbering of bit planes starts from 1, with the first bit plane corresponding to the least significant bit. Bit planes larger than 11 are not used due to significant signal distortions. For example, a change of one bit in the 11th bit plane corresponds to a signal change of 1024, resulting in a PSNR value falling below 20. Thus, embedding in the 11th bit plane produces substantial changes in the signal.

Figure 4 illustrates the results of two modifications for both baseline compression algorithms. The modifications of the same baseline method are represented with the same color and different line styles. The solid line depicts the results for methods with an adaptive selection of custom parameters. The custom parameter for the RLE algorithm is the number of bits B used for encoding the length of the bit sequence. For Huffman compression, the custom parameter is the length of one symbol L in the input bit stream. The adaptive method uses the optimal custom parameter value for each specific signal. After several experiments, we determined that the optimal values of B range from 2 to 10, while the optimal values of L correspond to a range of 2 to 8. The adaptive custom parameter selection modification of each compression method is based on testing all possible custom parameter values within the aforementioned ranges, followed by compression with the optimal custom parameter value. This adaptive custom parameter selection is computationally intensive.

To reduce the computational load, we analyzed the optimal custom parameter values for different numbers of bit planes to compress. We found that, in most cases, there is a strong relationship between the bit plane number and the optimal custom parameter value. As a result, we have determined the following empirical formulas for custom parameter selection:

(13) $$\begin{array}{l} B=P-3\;\left(for\;RLE\;compression\right),\\ L=P-2\;\left(for\;Huffman\;compression\right), \end{array}$$

where P is the compressed bit plane index. Experiments have shown that compression with custom parameters calculated by Formula (4) provides almost the same average space release compared to adaptive custom parameter selection. Figure 4 displays these results with dotted lines.

Figure 4 demonstrates that the lines corresponding to Huffman encoding are positioned above those corresponding to RLE from the 5th to the 9th bit plane. However, RLE shows an advantage for the 10th and 11th bit planes, notably only when B ranges from 2 to 10. Given that watermark embedding prioritizes lower bit planes to minimize distortion, Huffman encoding should be considered the superior choice over RLE for implementations that prioritize signal fidelity.

Regarding custom parameter selection, it is better to use empirical formulas than to rely on adaptive search. If there are no specific requirements regarding the distortion of a watermarked signal and the compression ratio, it is reasonable to select the 8th bit plane. A higher number of bit planes to compress corresponds to less growth in the saved storage space and a lower PSNR value. Embedding in several bit planes provides larger compression rates and increases watermark capacity, although the resulting distortion is determined by the highest bit plane used.

### 3.3. Performance Comparison

To provide an extensive comparison of the selected reversible watermarking algorithms, we tested all possible parameters for each algorithm. Ultimately, we selected the 15 best algorithm configurations for detailed comparison. Table 2 lists the selected best algorithm configurations. Below, we provide a brief explanation of the selected methods.

For LCB watermarking methods, we applied Huffman encoding because it outperforms RLE in all cases. Huffman encoding was implemented using adaptive length code symbol (L) selection. As mentioned in Section 3.2, the empirical Formula (13) can be used instead of adaptive search. The use of the empirical formula provides slightly less compression; however, the running time is significantly reduced. Additionally, we considered a modified LCB method based on prediction error compression (LCBP). In this method, the predicted signal is compressed, followed by watermark embedding.

For PEE watermarking, we tested (1) neighbor-based prediction using two neighbors flanking the predicted sample (Algorithm 6 in Table 1), (2) two inter-lead prediction variants (Algorithm 1 and Algorithm 2 in Table 1), and (3) physical-relationship prediction (Algorithm 3). File-specific regression models for inter-lead prediction outperformed the shared model trained on all files, motivating their selection for the final experiment. However, the shared-model approach remains viable in practice due to its lower computational costs while maintaining minimal quality degradation.

To compare the selected algorithms, we measured two main indicators of embedding efficiency: BPS and PSNR. We averaged these values over all files in the collection and across all twelve leads contained in each file (except for the physical prediction method, which is applicable only to the limb leads). BPS indicates how many watermark bits are embedded for each sample of the host signal, while PSNR illustrates the amount of distortion introduced into the host signal during the embedding process. A good algorithm should aim to maximize both values. It is evident that different practical scenarios may impose varying requirements on quality metrics; in particular, one metric may be significantly more important than the other. In the absence of specific requirements, it is reasonable to consider both metrics equally important.

**Figure 5:**
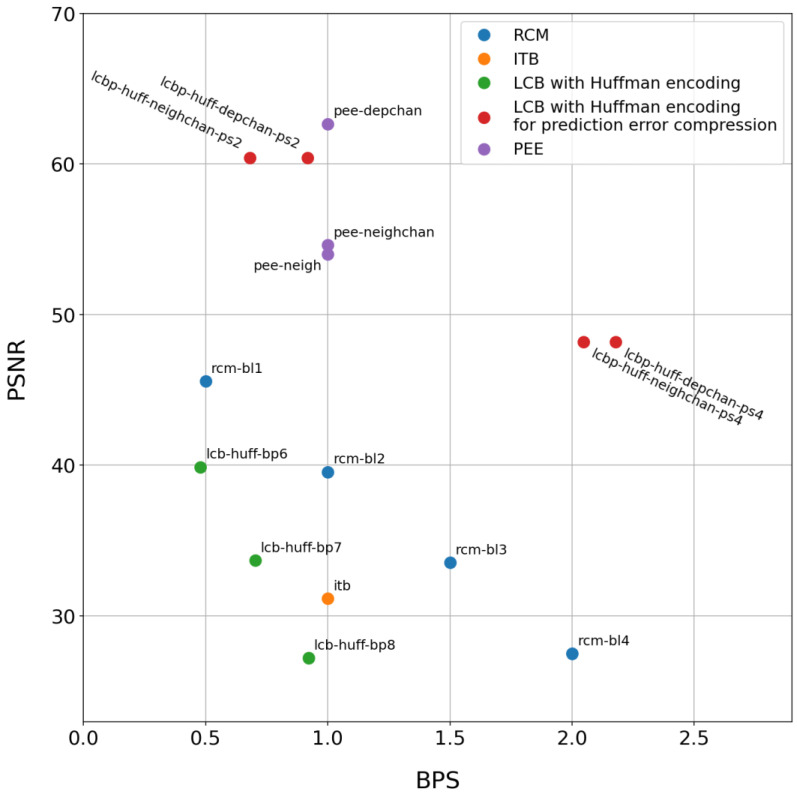
Reversible watermarking methods comparison.

The average BPS and PSNR of a watermarked signal obtained for the best algorithm configurations are shown in Figure 5.

The upper right corner corresponds to the best possible performance of the algorithms. It is evident from Figure 5 that LCB is the least preferable method because it has the lowest PSNR and BPS values. Among the LCB parameters, the best balance between quality metrics is achieved with a length of code symbol equal to 8. In terms of performance, the next method is ITB. It demonstrates higher performance than LCB but lower than RCM, PEE, and LCBP. However, the main advantage of ITB is its simple implementation without any parameters.

The middle area of the diagram in Figure 5 is occupied by RCM. This method has intermediate performance but provides a predictable BPS that can be varied within a wide range. Additionally, it allows embedding in all leads simultaneously. One of the best methods is LCBP; it can be used when high PSNR or high BPS is required, depending on the method’s modification. However, LCBP depends on prediction methods and requires embedding into leads that have a linear relationship. Another effective method is PEE, which exhibits predictable BPS and high PSNR values. RCM demonstrates linear growth in watermark capacity with the increase in the bit length parameter.

The evaluated watermarking schemes achieve high-quality ECG signal reconstruction while introducing minimal distortions (as measured by PSNR) and maintaining substantial watermark capacity. All algorithms feature straightforward implementations, with the PEE and LCBP methods offering an optimal balance between watermark payload capacity and watermarked signal quality.

**Figure 6:**
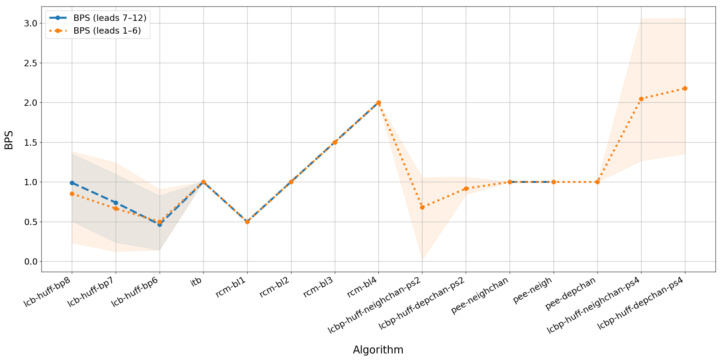
Average BPS for selected algorithms depending on lead group.

**Figure 7:**
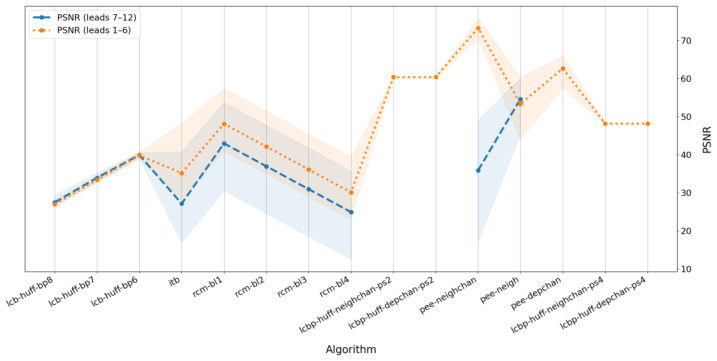
Average PSNR for selected algorithms depending on lead group.

To conduct a comparative investigation of the different algorithms, we measured BPS and PSNR values independently for two groups of leads. The first group comprises six limb leads: I, II, III, aVR, aVL, and aVF. The second group consists of six precordial leads (V_1_–V_6_). The results are presented in Figure 6 and Figure 7. As in Figure 3 and Figure 4, the colored backgrounds represent the dispersion of values relative to the mean values shown by the lines.

Figure 6 shows the difference in watermark capacity between two groups of leads, while Figure 7 illustrates the difference in the degree of distortion in the watermarked signal across these groups. The blue line (representing precordial leads) is omitted for algorithms that use physical-based prediction because these methods are applicable only to limb leads, as described in (10). Additionally, for low prediction quality (LCBP with regression), the blue line is absent because successful watermark embedding could not be achieved in that case.

Figure 6 indicates that the BPS values are nearly identical for the two groups of leads, with a slight difference appearing only for the LCB method when using high bit planes. In contrast, Figure 7 demonstrates that the average PSNR differs significantly for several algorithms, depending on the lead group. Specifically, for the group of precordial leads, the degree of distortion is higher than that observed for the limb leads, with the only exception being the LCB algorithms without prediction, where the group of leads influences BPS rather than PSNR.

Overall, the quality of watermark embedding in signals derived from limb leads is generally higher than in those from precordial leads, and some algorithms are not applicable to the precordial lead group at all.

Additionally, we must mention the robustness of the considered algorithms. Although a quantitative study of this indicator was not conducted in this work, some basic assumptions can be made based on our understanding of the mechanics of embedding algorithms.

All methods discussed in this paper create fragile or semi-fragile digital watermarks. Compression-based methods change specific bit planes, so watermarked signals created by them are resistant to distortion of bits that remain unchanged by malicious modifications. The remaining methods affect several low-order bits, the number of which is not fixed and depends on many factors for each sample.

The watermarks can be made more robust by using redundant embedding or correction codes; however, this method may not always be effective. Thus, the watermarked signals obtained by methods based on compression are very sensitive to distortion, as even a change in one bit of compressed information can completely alter the result of decompression or render it impossible altogether. The remaining algorithms are more resistant to minor distortions but come with some caveats. For example, in ITB, one of the samples contains a parity bit used to decode the entire signal. Changing this bit will result in a complete inversion of the extracted bits. At the same time, RCM requires precise synchronization when using a mode that allows skipping samples that are not suitable for embedding. If this mode is used, then changing one bit can lead to the destruction of the entire watermark.

### 3.4. Final Outcomes

Now let us summarize the main outcomes from the experimental investigations.

The most crucial part of the PEE watermarking method is the prediction algorithm. We demonstrated that for limb leads, the best signal prediction corresponds to the linear regression method, while for precordial leads, neighbor-based prediction yields better results. The specific formula for neighbor-based prediction is provided in the text of the article. We considered two approaches to estimating regression coefficients: one is learning from a single file, and the other is learning from a group of files. The first method gives the best prediction error, while the second method is better in terms of computational load. The last prediction method we discussed is physical-based prediction, which relies on the physical dependence of limb leads. This method is slightly less effective than regression in terms of prediction error; however, it is simpler to implement.

For LCB compression, it is essential to determine which bit plane of the signal to compress and what type of lossless compression algorithm to use. We tested run-length encoding (RLE) and Huffman encoding with different variants of bit planes for compression. It was found that Huffman encoding provides a better compression ratio than RLE. A better compression ratio corresponds to improved watermark capacity. In the text of the article, we proposed empirical formulas for calculating suitable RLE and Huffman encoding parameters. The use of these formulas simplifies the process of algorithm parameter selection. Moreover, we considered a modification of the LCB approach where prediction error was compressed instead of the actual signal. The research has shown that LCBP (LCB with prediction error compression) is more effective in terms of compression ratio than the standard LCB approach.

Finally, we tested all possible variants of algorithm implementations and provided a detailed comparison of the 15 best algorithm configurations. It was found that the best approaches to reversible ECG watermarking are PEE and LCBP. Both methods yield the highest values of watermark capacity and PSNR among the others. The lowest PSNR and BPS values correspond to the LCB approach, while RCM showed intermediate results.

The watermarking schemes considered can balance the capacity of the watermark while achieving a high-quality restored ECG signal with no perceptible distortion. Furthermore, all algorithms are easy to implement, with PEE and LCBP providing the best trade-off between watermark capacity and the quality of the watermarked signal.

## 4. Conclusions

In this paper, we provide an extensive comparison of four reversible watermarking methods for ECG data protection. The methods considered in our research are reversible contrast mapping difference expansion (RCM), integer transform-based difference expansion (ITB), prediction error expansion (PEE), and lossless compression-based embedding (LCB). Most of these methods have been originally developed for image watermarking and have not been tested with ECG data yet, except for the PEE approach. Using our own implementation of the aforementioned algorithms and ECG file subset from the PhysioNet database, we found the best parameter settings for all algorithms and compared watermark capacity and imperceptibility characteristics.

In the course of a comparative analysis, the distinctive features of each reviewed method were identified, and areas of their application were proposed:

- If minimizing container distortion is critical, one should use PEE algorithms (with prediction based on adjacent samples or linear-dependence formulas) or LCBP (featuring Huffman coding and a low number of modified bit planes). This situation may arise, for instance, when a physician performs a preliminary ECG analysis before extracting the watermark and restoring the signal’s original appearance.
- If a high and predictable embedding capacity is more important, applying the RCM-DE method with a larger number of bits per pair of container samples or the ITB-DE algorithm is advisable. A significant embedding capacity might be required, for example, if error-correcting coding is implemented to ensure accurate container recovery even after any distortions or interference during file transmission or storage.
- If a compromise is needed, then LCBP with Huffman coding and a higher number of modified bit planes should be used. However, if simplicity of implementation and low computational complexity are prioritized, as well as the option to embed in all channels simultaneously, one can opt for ITB-DE or RCM-DE with a smaller number of bits per pair of container samples

Many modern ECG monitoring systems employ reduced-lead configurations, with some devices using only one or two leads to balance diagnostic efficacy with wearability and ease of use [26,27]. When implementing digital watermarking techniques for these reduced-lead ECG measurements, the available methodological options are more constrained compared to 12-lead signals. Specifically, for 1- and 2-lead signals, algorithms based on physical prediction (designated as “depchan”) cannot be applied. In addition, for single-lead signals, we cannot use inter-lead prediction methods (designated as “neighchan”). However, all other algorithms considered in this paper can be successfully applied.

The data presented in this study are openly available in [28].

## Figures and Tables

**Table 1:** Evaluated prediction algorithms.

#	Notation	Description
1	Regression by file	Training a separate regression model for each ECG file, where one lead is used for prediction and the remaining leads are used for model training
2	Regression by collection of files	Training a common regression model for a collection of ECG files, where one lead is used for prediction and the remaining leads are used for model training
3	Physical prediction	Physical prediction using formulas from Section 2.7.3
4	Neighbor averaging (1,0) or (0,1)	$\hat{x_{i}}=y_{i-1}^{w}$ or $\hat{x_{i}}=x_{i+1}$
5	Neighbor averaging (2,0) or (0,2)	$\hat{x_{i}}=\frac{y_{i-2}^{w}+y_{i-1}^{w}}{2}$ or $\hat{x_{i}}=\frac{x_{i+1}+x_{i+2}}{2}$
6	Neighbor averaging (1,1)	$\hat{x_{i}}=\frac{y_{i-1}^{w}+x_{i+1}}{2}$
7	Neighbor averaging (2,2)	$\hat{x_{i}}=\frac{y_{i-2}^{w}+y_{i-1}^{w}+x_{i+1}+x_{i+2}}{4}$

**Table 2:** Best algorithms’ configurations.

Notation	Embedding Method	Prediction Method	Compression Method	Parameters Used
lcb-huff-bp8 lcb-huff-bp7 lcb-huff-bp6	LCB	-	Huffman	The number after the letters “bp” indicates the number of the lower of the two successive bit planes that were compressed and overwritten. Obviously, in practice, the bit planes have to be chosen based on the required balance between BPS and PSNR.
itb	ITB	-	-	Method has no adjustable parameters
rcm-bl1 rcm-bl2 rcm-bl3 rcm-bl4	RCM	-	-	The number after the letters “bl” means the number of digital signal bits built into one pair of host signal samples. In this case, BPS is equal to half of this number
lcbp-huff-neighchan-ps2	LCBP	neighchan *		The suffix “ps2” means that two bit planes are used to record the compressed prediction errors. To minimize distortion, it is reasonable to use the least significant bit planes.
lcbp-huff-depchan-ps2		depchan *	Huffman	
lcbp-huff-depchan-ps4		depchan *		
pee-neighchan	PEE	neighchan *		These combinations differ in prediction methods. The neighbor prediction algorithm is used only here because it is not applicable to the prediction error compression method (LCBP).
pee-neigh		neigh *	-	
pee-depchan		depchan *		
lcbp-huff-neighchan-ps4	LCBP	neighchan *	Huffman	The “ps4” suffix means that four bit planes are used to record the compressed prediction errors, which significantly increases the embedding volume compared to the two-plane option.
lcbp-huff-depchan-ps4		depchan *		

* The following short notations for prediction algorithms are used: “neigh” is neighbor-based prediction with two neighbors from both sides of predicted sample, “neighchan” is inter-lead prediction, and “depchan” is physical-based prediction.

## Data Availability

The data used in the study are openly available in PhysioNet project database [25]. The source code of the watermarking algorithms is openly available in [28].
